# MEPT-LLM: a multimodal generative AI model for identifying and understanding cultural-emotional barriers in the language classroom

**DOI:** 10.3389/fpsyg.2026.1759927

**Published:** 2026-06-03

**Authors:** Xiaoli Bai, Meixian Bai

**Affiliations:** 1 International College, Southwest University, Chongqing, China; 2 School of Foreign Languages, Neijiang Normal University, Sichuan, China

**Keywords:** cross-cultural education, cultural adaptation, emotional barriers, intelligent education, personalized generation, psychological state

## Abstract

With the rapid transformation of the educational environment driven by artificial intelligence (AI), cross-cultural communication in teaching Chinese as a foreign language has seen significant advancements. However, traditional models still face limitations in emotion recognition, content generation, and personalized recommendations, which restrict their effectiveness in multimodal educational settings, particularly when addressing emotional barriers in language classrooms. To overcome these challenges, this paper proposes the MEPT-LLM model, which integrates three core modules: multimodal emotion perception, psychological state analysis, and personalized content generation. Experimental results demonstrate that MEPT-LLM outperforms traditional models on the MELD and MEMD datasets, with improvements in emotion perception, content generation quality, and personalized recommendations. Specifically, the model achieves a performance increase of 12%-15%. Ablation experiments further highlight the crucial role of each module, with the collaborative effect of the modules being key to the model's success. The MEPT-LLM model provides strong support for the development of intelligent learning systems in cross-cultural education, particularly in educational psychology, by optimizing learning motivation through emotional regulation and personalized feedback.

## 1 Introduction

The rapid development of globalization and information technology has brought significant transformations to the education sector, especially in the field of language learning. As students engage in cross-cultural language learning (Parkinson, 2023; Du et al., 2025), the challenges posed by cultural differences and emotional barriers have become prominent obstacles, affecting their participation, engagement, and motivation (Wang et al., 2024). Learners' emotional states are shaped not only by the complexities of language comprehension but also by difficulties in cultural adaptation and the dynamics of interaction. These cultural-emotional barriers, refer to the emotional and cultural challenges that learners face in the classroom, particularly when dealing with issues such as foreign language anxiety, acculturation stress, stereotype threat, and communication apprehension (Zhou et al., 2025). These barriers manifest not only in language skills, such as speech and grammar, but also in aspects like classroom participation, learning strategy choices, and emotional expression. Therefore, the identification and regulation of students' emotional states, particularly overcoming cultural-emotional barriers, has become a central focus for improving the effectiveness of teaching and enhancing students' learning motivation. Addressing these challenges is critical to fostering a more inclusive, engaging, and effective learning environment, where learners can thrive despite cultural and emotional challenges (Cheng et al., 2024b; Ullah, 2024).

In recent years, with the rapid development of artificial intelligence technologies, particularly deep learning, various intelligent teaching systems have gradually been introduced into the education sector. Emotion computing, a technology aimed at enhancing learning outcomes by recognizing and regulating students' emotional states, has been widely applied (Lu et al., 2025). CNN-based emotion recognition methods have been extensively used in facial expression analysis and emotion classification (Flower and Jaya, 2024). Emotion analysis models based on LSTM have also had considerable success in classifying textual feelings (Huang et al., 2021). SVM-based methods are used in the analysis of linguistic emotions, particularly in the recognition of linguistic emotions (Kumar et al., 2023). GNN-based methods of emotion analysis model the transmission and influence of emotions using graph structures, making them suitable for complex social contexts (Wang and Kim, 2024). Emotion Labeling Systems (ELS) automatically record and classify students' emotional states, which greatly improves recognition and analysis of emotions (Lian et al., 2023). Despite the In view of the effectiveness of these technologies in recognizing students emotional states, existing models often do not take into account the Impact of cultural differences on student health emotional expression and perception (Ali et al., 2026).

The methods of Multimodal education has developed rapidly in the field of education. The researchers tried to improve the accuracy of emotion recognition and adaptability of patterns using different means of information such as visuals, a combination of language and text (Bi and Su, 2025). The visual-speech fusion network (VGG-16+RNN) was used for the joint modeling of emotions emotional analysis is performed by integrating facial expressions and voice signals (Wang et al., 2023). Transformer-based multimodal fusion models leverage the self-attention mechanism to capture emotional features from both text and speech information, successfully applied to cross-modal emotion analysis tasks (Cheng et al., 2024b). In addition to the combination of speech and images, emotion modeling based on deep autoencoders (Deep Autoencoder) extracts features from image and text data to implement multimodal learning (Cheng et al., 2024a). In recent years, there has been an increasing amount of research using deep collaborative filtering algorithms (Deep Collaborative Filtering) to handle multimodal emotional data, particularly in recommendation systems for recommending emotion-related content to users (Karabila et al., 2023). Methods based on RL that combine images and text data, are successfully applied in the analysis of emotions and personalized recommendations (Govea et al., 2024). Despite positive progress in recognizing multimodal emotions in some educational scenarios, limitations exist due to the complex connection between emotional states students and their cultural background, in particular in the practical application of cultural adaptation and the creation of personalized content (Rajput et al., 2025).

In addition, research into the cultural regulation of emotions has attracted increasing attention, particularly in the fields of cultural education and mental health interventions. Various approaches, including those based on deep learning, have been proposed to regulate emotions across different cultures. These approaches often include models of cultural adaptation, which analyze students' cultural content and adapt educational content and interaction (Fekar Gharamaleki and Fathipour-Azar, 2025) Ning et al. (2026). To improve the learning experience and emotional responses, BERT-based models have been used to address emotional regulation across languages, promoting motivation in students from different cultures (Li and Washington, 2024). Emotional Reinforcement Learning (ERL) has been implemented in educational institutions to create personalized learning strategies for students with different emotional states (Govea et al., 2024). Another approach uses conditional GANs, which use generative models to create content specifically tailored to cultural contexts (Srivastava, 2025). Methods of transfer education, particularly in intercultural education, use existing emotional models to better meet the needs of students from different cultures (Padi et al., 2021). Self-Supervised Learning (SSL) has been integrated into emotional regulation systems to improve the effectiveness of cultural and emotional regulation tasks through unsupervised teaching methods. Despite this progress, challenges remain in areas such as multimodal analysis of emotions and real-time personalized content generation, especially when considering the practical feasibility and effectiveness of applying these methods in real educational settings.

Despite some progress in analyzing emotions and cultural adaptation using the methods mentioned above, existing studies have pointed out that emotional and cultural barriers have not yet been fully addressed, especially in the specific context of language classrooms. The main objective of this article is to develop and introduce a new multimodal generative AI model, MEPT-LLM, to overcome these cultural and emotional barriers in language classes. By integrating multimodal emotion recognition technology and combining emotion regulation with cultural context modeling, this paper provides a comprehensive foundation for generating educational content. The model allows for the accurate perception and real-time correction of students' emotional states. Three key contributions to this document:

- A multimodal emotion perception module has been introduced that combines ResNet50 and ViT and improves the accuracy of emotional state recognition.
- The centralized module of analysis of Psychological state integrates basic emotional and cultural information and provides a solid foundation for the creation of personalized educational content.
- A psychoadaptor is being developed that uses LoRA technology to dynamically adapt the generated content, to overcome cultural and emotional barriers and increase learning motivation.

## 2 Theory method

### 2.1 Datasets

In the experimental section of this study, we utilized two publicly available multimodal emotion recognition datasets: MELD (Ma et al., 2023) and MEMD (Liang et al., 2024). The MELD dataset contains text, audio, and video modes and is primarily designed for emotion recognition in dialogues, making it suitable for the analysis of emotional traits such as language and facial expressions. This aligns well with the analysis of emotional behaviors in educational contexts, particularly language classroom interactions, where both verbal and non-verbal emotional cues are important. The MEMD dataset, which encompasses audio, video, and physiological signals, provides a more comprehensive approach to multimodal emotional analysis. It is particularly valuable for understanding emotional states in complex educational settings, where multiple modalities can offer a more holistic view of learners' emotional responses. Additionally, MEMD offers advanced emotion annotations, supporting a wide range of emotion recognition tasks that are essential for our study. Table 1 describes the basic characteristics of these data sets, including comparison of their modalities, categories of emotions, corpus sizes, and their applicability.

**Table 1:** Comparison of key characteristics between MELD and MEMD datasets.

Dataset	Modalities	Emotion categories	Corpus size	Applicability
MELD	Text + Audio + Video	Anger, Disgust, Sadness, Joy, Neutral, Surprise, Fear	About 13,000 dialogue sentences	Suitable for multimodal emotion recognition, especially speech, facial expression analysis
MEMD	Audio + Video + Physiological Signals	Multiple Emotion Categories	Data from about 250 participants	Suitable for emotion recognition with rich multimodal data

At the pre-processing stage, we first cleaned and organized the MELD dataset. Each dialogue in the dataset was labeled with different categories of emotions, including anger, aversion, sadness, happiness, neutrality, surprise, and fear. We tokenized the textual data and standardized emotional labels to match the emotion classification system used in our experiments. For the audio data, we performed feature extraction focusing on Mel spectrogram characteristics, which were then used for emotion analysis. For video data, we conducted facial expression recognition at key points and extracted expression-related features using the ResNet50 model.

Similarly, we applied the same pre-processing steps for MEMD. This dataset includes audio, video, and physiological signals categorized into several emotions. The audio signals were processed to quantitatively determine emotional states by extracting Mel spectrograms and other relevant characteristics. Video data was extracted from key points of facial expression, while physiological signals, including EEG data, were preprocessed to extract emotional traits. We standardized the data to ensure it is ready to be fed into a model for learning and testing. Although MEMD was not originally designed for language classroom interactions or cross-cultural dynamics, its multimodal nature (audio, video, and physiological data) makes it a valuable resource for emotion recognition in educational contexts.

### 2.2 Experimental details

In this study, to ensure the reproducibility and reliability of the experiments, all tests were conducted in a consistent hardware and software environment. The experiments utilized two NVIDIA A100 40GB GPUs, with 256GB of RAM and an NVMe SSD RAID 0 array to provide fast data access and processing during training. The operating system used was Ubuntu 22.04 LTS, and the deep learning framework was PyTorch 2.1.0 with CUDA 12.2. For the multimodal emotion analysis task, pre-trained models based on ResNet50 and ViT were used to extract features from images and videos, while audio features were represented using Mel-spectrograms, ensuring effective feature extraction when combining multimodal data. In terms of data preprocessing, standard tokenization tools were used, and text data underwent denoising and cleaning. Audio and video data were also standardized appropriately to ensure the quality of the input data. Additionally, to improve emotion recognition performance, data augmentation techniques, including synonym replacement, noise addition, and facial expression variation simulation, were applied.

During the experiment, we performed several training and validation checks to ensure the stability and effectiveness of the results. The AdamW optimizer with an adaptive learning rate scheduling strategy was employed during training to facilitate efficient model convergence. We carefully tuned the model's hyperparameters: the batch size was set to 32, and the initial learning rate was set to 1e-4 using the AdamW optimizer, with adjustments being made through an adaptive learning rate strategy. The learning rate was reduced by a factor of 0.1 every 10 epochs to ensure stable model convergence. A total of 50 training epochs were conducted, with the optimal number of epochs being selected through cross-validation. Additionally, we performed fine-tuning based on the model's performance on the validation set, and these configurations were implemented to ensure model stability and efficiency. In the multi-task learning experiments, the model simultaneously performed emotion classification, emotion intensity prediction, and emotion recognition tasks.

### 2.3 Evaluation metrics

In the experimental evaluation of this study, we employed five common evaluation methods to comprehensively assess the model's performance. Through the combined evaluation of these methods, we were able to analyze the model's performance in emotion recognition and personalized content generation tasks from multiple perspectives. Each method provides valuable insights into the model's accuracy, generation quality, diversity, and the alignment of emotional ranking, ensuring both the effectiveness and practicality of the model (Younis et al., 2024).

BLEU (Bilingual Evaluation Understudy) is commonly used to evaluate the similarity between generated text and reference text, making it particularly suitable for machine translation tasks. However, it has also proven to be effective in emotion analysis and content generation. In our study, BLEU is used to assess the emotional and cultural suitability of the generated educational responses. Specifically, *p*_*n*_ represents the n-gram precision, which is the number of n-grams in the generated text that match the reference text, divided by the total number of n-grams in the generated text. *w*_*n*_ is the n-gram weight, typically set to *N* = 4 when $w_{n}=\frac{1}{4}$. BP is the brevity penalty factor, used to penalize overly short generated texts. This metric is used to assess the degree of alignment between the generated emotional content and the reference content, capturing both linguistic accuracy and emotional relevance. The higher the BLEU score, the more similar the generated content is to the reference content, indicating a better emotional and cultural fit. We validated BLEU scores by comparing them with human ratings of content quality and emotional appropriateness, showing a strong correlation between high BLEU scores and human evaluations.


$$\begin{array}{l} \text{BLEU}=BP\cdot \exp\left(\overset{N}{\underset{n=1}{\sum }}w_{n}\log p_{n}\right) \end{array}$$ (1)

Macro-F1 is used to evaluate model performance in all categories for multi-class tasks. k is the total number of emotion categories and *F*1_*i*_ is the point F1 for the i-th emotion category. The F1 score is calculated as the average harmonic accuracy and memory. Calculating the F1 score for each category and the average balances the impact of each category on model performance, which makes it particularly useful for analyzing emotions with unbalanced categories.


$$\begin{array}{l} \text{Macro}-\text{F}1=\frac{1}{k}\overset{k}{\underset{i=1}{\sum }}F1_{i} \end{array}$$ (2)

The F1 score is an extension of the Macro-F1 Score, which takes into account the share of samples in each category and effectively solves the problem of class imbalance. *w*_*i*_ represents the part of the sample for category i of the test set and *F*1_*i*_ represents the F1 score for category i. Thanks to the weighting the F1 scores, this indicator better reflects the overall performance of the model in the absence of class inequalities.


$$\begin{array}{l} \text{Weighted-F}1=\overset{k}{\underset{i=1}{\sum }}\left(w_{i}\cdot F1_{i}\right) \end{array}$$ (3)

Normalized Discounted Cumulative Gain (nDCG) is used to evaluate the ranking performance of generated content, and it is widely applied in emotion analysis and recommendation systems. In this study, *p* represents the length of the result list considered, *rel*_*i*_ is the relevance score of the result at the i-th position, and *IDCG*_*p*_ is the ideal DCG, which represents the DCG when the results are sorted in descending order based on true relevance. The nDCG metric is used to evaluate how well the generated content matches the learner's emotional and cultural context. The higher the nDCG value, the closer the generated content's ranking is to the ideal ranking, indicating a higher alignment between the emotional content and the learner's true needs.


$$\begin{array}{l} \text{DC}{\text{G}}_{p}=\overset{p}{\underset{i=1}{\sum }}\frac{2^{\text{re}{\text{l}}_{i}}-1}{\log_{2}\left(i+1\right)} \end{array}$$ (4)

$$\begin{array}{l} \text{nDC}{\text{G}}_{p}=\frac{\text{DC}{\text{G}}_{p}}{\text{IDC}{\text{G}}_{p}} \end{array}$$ (5)

Dist-n is used to evaluate the diversity of generated text, particularly the diversity of vocabulary and phrases. *n* typically takes the values of 1 or 2, assessing vocabulary diversity (Dist-1) and phrase diversity (Dist-2), respectively.


$$\begin{array}{l} \text{Dist-n}=\frac{\left|\text{unique n-grams in generated text}\right|}{\left|\text{total n-grams in generated text}\right|} \end{array}$$ (6)

Through these evaluation metrics, we are able to comprehensively analyze the performance of the MEPT-LLM model in multimodal emotion recognition and personalized content generation tasks from different perspectives, ensuring its effectiveness and applicability in emotion understanding and generation tasks.

## 3 Numerical model

### 3.1 Overview of our network

**Figure 1:**
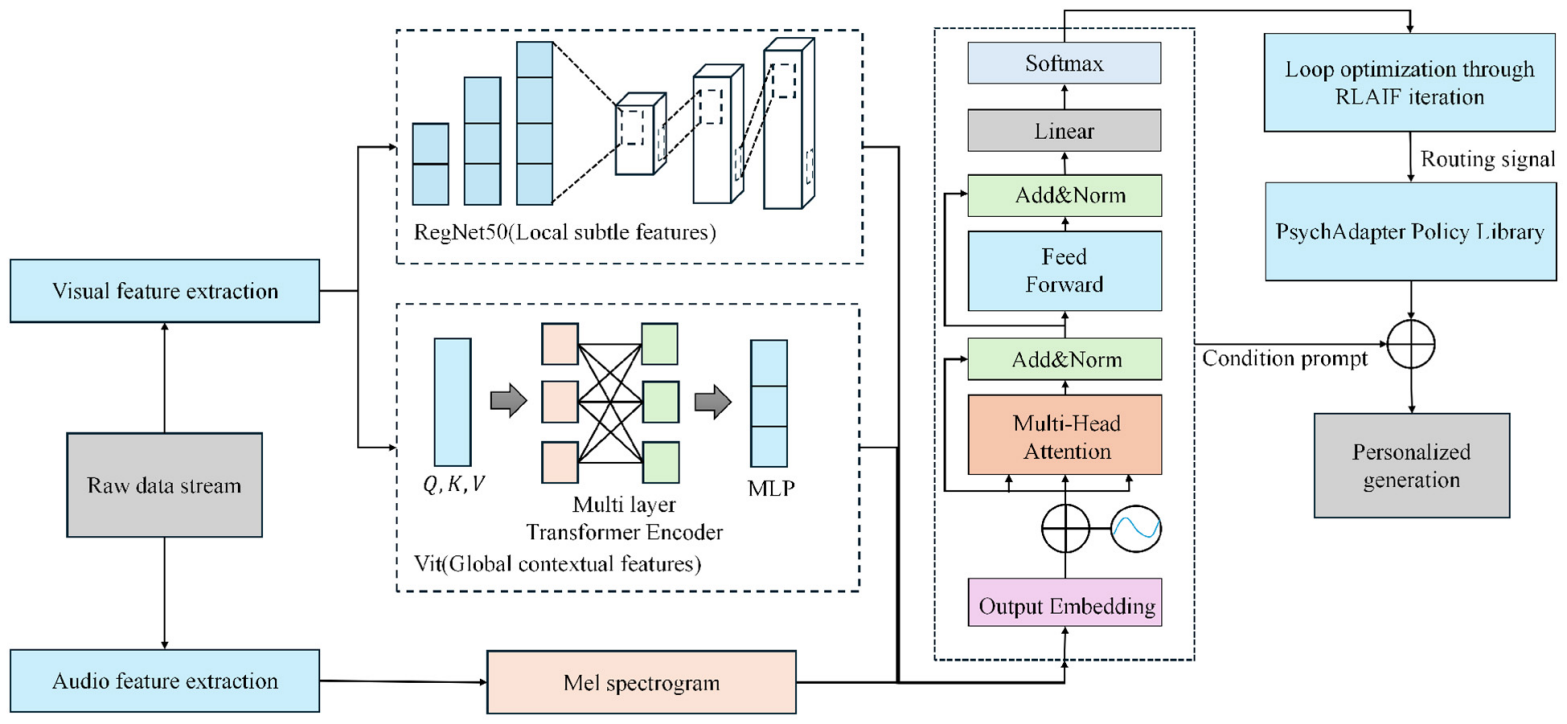
Architecture of the MEPT-LLM model for overcoming cultural-emotional barriers in the language classroom.

The MEPT-LLM model proposed in this paper is based on the Transformer architecture and aims to overcome cultural emotional barriers in language classes. including technologies to detect multimodal emotions and generate personalized content. The central structure of the model consists of a multimodal module for the perception of emotions, a central module for the analysis of the psychological state and a module for the generation and interaction of personalized content. These modules work in close coordination and form the entire process from recognizing emotions to generating personalized responses. By transmitting and transforming the data flow, the model offers high efficiency and precision in managing complex emotional states. Figure 1 shows the general structure of the model.

In our framework, we distinguish between “emotional state” and “psychological state” to better understand the learner's mental and emotional conditions. The “emotional state” specifically refers to the learner's current emotional responses, such as anxiety, joy, frustration, or excitement. These emotional responses are directly influenced by situational factors such as classroom interactions, learning challenges, or external stressors. Emotional states are typically transient and can fluctuate rapidly based on changes in the environment or specific learning experiences. In contrast, the “psychological state” is a broader and more comprehensive construct that encompasses not only the emotional state but also cognitive factors, such as cognitive load, motivation, and cultural adaptation. The psychological state represents the learner's overall mental condition and is generally more stable than the emotional state. It reflects the learner's sustained well-being and learning conditions, providing a holistic understanding of their emotional, cognitive, and cultural adaptation status. This distinction allows for more personalized and adaptive educational responses, addressing the learner's needs across both emotional and cognitive dimensions. Specifically, recognizing a learner's emotional state enables the system to adjust the difficulty level of tasks, provide emotional support, or modify the instructional approach to increase engagement and motivation. This emotional recognition is crucial in creating a personalized learning environment that enhances learning outcomes. We have strengthened this argument by providing evidence from previous studies that demonstrate the positive impact of emotionally adaptive learning environments on student outcomes, such as increased motivation, engagement, and academic performance. This personalization not only improves the immediate learning experience but also contributes to long-term academic success and well-being.

Moreover, emotional recognition alone is not sufficient to improve learning outcomes. The integration of pedagogical practices with emotional insights is crucial for enhancing educational effectiveness. In our framework, emotional recognition is combined with personalized learning interventions, allowing the system to tailor its approach based on the learner's emotional and cognitive state. For instance, if the system identifies a learner as being anxious, it may reduce the task difficulty, offer emotional encouragement, or adapt teaching strategies to better align with the learner's current emotional state. These interventions are not only designed to address emotional barriers but also to foster a more engaging and motivating learning environment. By integrating emotional recognition with personalized pedagogical practices, we create a dynamic learning experience that can significantly improve student engagement, motivation, and retention, ultimately leading to better learning outcomes.

The multimodal emotion perception module serves as the input layer of the model, responsible for extracting emotional characteristics from the learner's multimodal data (Venkatraman et al., 2024) Ning et al. (2026). This module integrates three modes—visual, audio, and text—using heterogeneous networks such as ResNet50 and ViT to extract facial expressions and overall contextual information. The Mel spectrogram is used to process linguistic data, and these characteristics eventually merge into the multimodal vector of emotional characteristics. These extracted features are then used as input for the following modules and provide essential data for the emotional analysis and decision-making processes of the model. The centralized psychological state analysis module receives feature vectors from the multimodal emotion perception module and is designed to deeply merge and analyze this data (Hazmoune and Bougamouza, 2024). This module integrates the functions of the different modes using a multimodal fusion transformer, analyzing the character and intensity of emotions, as well as the cognitive load and cultural trends of the learner. The aggregated information is then transformed by the state encoder into a structured vector of the learner's psychological state, allowing us to quantify their emotional and cognitive state. This psychological state vector serves as the primary signal that drives the subsequent generation of personalized content. The personalized content generation and interaction module generates specific teaching content based on the psychological state vector, ensuring that the generated content meets the emotional needs of the learner while being appropriately adjusted according to their cultural background (Yu et al., 2024). This module uses a basic language model and the PsychAdapter strategy library, optimized with LoRA technology. It selects the most suitable adapter depending on the psychological state vector and dynamically adjusts the generation of educational content based on different emotional and cultural contexts. Ultimately, the module generates personalized content such as dialogues, comments, and practical exercises, helping students overcome emotional barriers and improving their motivation to learn.

The MEPT-LLM model enables a seamless transition from emotion perception to personalized content generation, thanks to the effective collaboration of its three modules. Each module plays a decisive role in the model's effectiveness and accuracy in regulating emotions and adapting to cultural needs. With these integrated components, MEPT-LLM excels not only in traditional emotion recognition tasks but also in dynamically generating learning content tailored to the emotional needs and cultural background of the student. This model proposes an innovative solution, encouraging the broader application of educational technologies in emotion regulation and personalized learning.

### 3.2 Multimodal emotion perception module in emotion recognition systems

**Figure 2:**
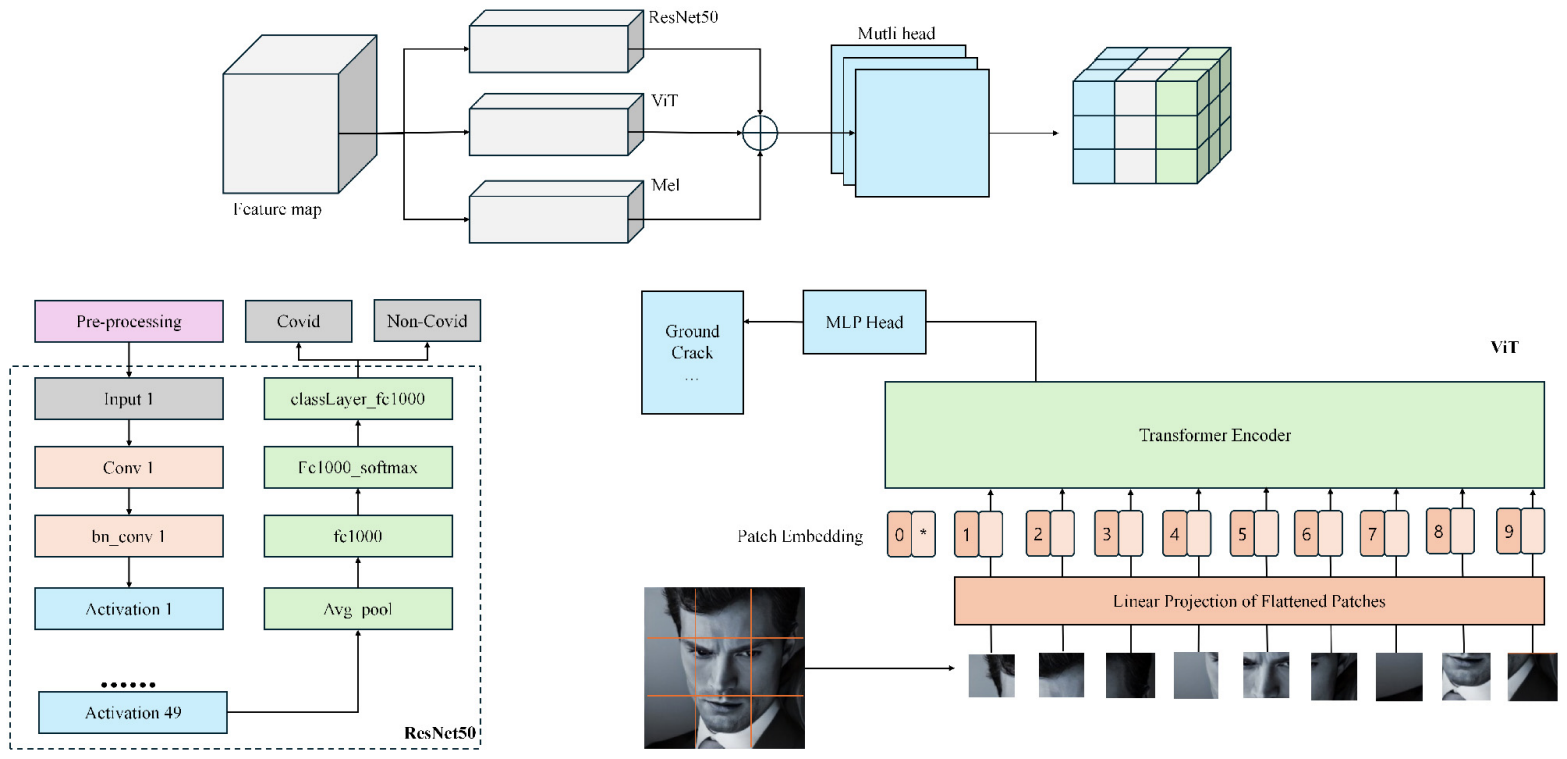
Architecture of the multimodal emotion perception module using ResNet50, ViT, and Mel-spectrogram for emotion recognition.

The Multimodal Emotion Perception module serves as the input layer to the MEPT-LLM model, responsible for extracting emotion-related features from the student's multimodal data. This module combines data from three modes—visual, audio, and text—using heterogeneous networks such as ResNet50 and ViT to extract facial expressions and overall contextual information. Audio data is processed through Mel spectrograms, and these features are integrated into a vector representing multimodal emotional characteristics. The extracted characteristics are then used as inputs to the subsequent modules, providing baseline data for analyzing the model's emotion recognition and decision-making processes. Figure 2 shows the overall structure of this module.

In the visual feature extraction section, ResNet50 extracts the characteristics of the facial expression through the rest of the learning structure. The Residual learning helps the models solve the problem of the disappearing gradient in deep networks, by enabling networks to capture deeper and more complex visual characteristics. *x*_*l*_ is the entrance of the l-th residual block, and *F*(*x*_*l*_, {*W*_*l*_}) is the mapping of the weight obtained within the residual block, typically from the layers of the breakdown, the batch normalization and activation functions of the ReLU. *y*_*l*_ is the output, and the representation of the identity mapping (shortcut connection) ensures an effective formation of the network. This process ensures accurate detection of fine changes in facial expression, such as frowning or smiling.


$$\begin{array}{l} y_{l}=F\left(x_{l},\left\{W_{l}\right\}\right)+x_{l} \end{array}$$ (7)

ViT collects global contextual information and continues to extract the emotional characteristics of the student by integrating patches and a self-attention mechanism. *x*_*pi*_ represents the i-th image patch, *E* is the projection teaching matrix, *E*_*pos*_ is the positional encoding and *z*_0_ is the processed image of the sequence. Through these processes, ViT efficiently extracts overall information from the image and provides overall emotional indications to the emotion perception module.


$$\begin{array}{l} z_{0}=\left[x_{class};x_{p1}^{E};x_{p2}^{E};\cdots \;;x_{pN}^{E}\right]+E_{pos} \end{array}$$ (8)

In the audio feature extraction section, we convert the audio signal into a time-frequency spectrogram using Mel-spectrograms, thereby extracting emotional features. *x* represents the audio signal, *w*[*k*] is the window function, and *M*[*m, n*] is the value of the Mel-spectrogram at position (*m, n*). Through this process, we are able to transform the audio signal into a time-frequency representation that reflects human auditory perception, providing effective audio features for subsequent emotion analysis.


$$\begin{array}{l} M\left[m,n\right]=\log\left(|\overset{N-1}{\underset{k=0}{\sum }}w\left[k\right]\cdot x\left[n\cdot h+k\right]\cdot e^{-j2\pi km/M}{|}^{2}\right) \end{array}$$ (9)

Text data is converted into vector images by integrating words and encoding positions. The vector representation of each word includes both the semantic information of the word and the order of its position. *w*_*i*_ is the i-th word of the sequence, *E*_*word*_ is the position encoding matrix, *E*_*pos*_ is the matrix of coding the position and $h_{i}^{0}$ is the original representation of the word entered into the first layer of the Transformer. Thus, text data effectively represents semantic information, while the information is stored in word order.


$$\begin{array}{l} h_{i}^{0}=E_{word}\left[w_{i}\right]+E_{pos}\left[i\right] \end{array}$$ (10)

To further improve the model's perception of emotions, the multimodal aggregation of functions is carried out through a weighted attention grouping mechanism. *h*_*i*_ is the vector of the i-th modality, α_*i*_ is the corresponding attention weight and *v* is the definitively generated vector of the solid-dimensional characteristics. By weighing the vectors of each model's characteristics, the model can focus on the most important emotional information, while excess parts are ignored.


$$\begin{array}{l} \alpha_{i}=\frac{\exp\left(w^{T}\tanh\left(Wh_{j}\right)\right)}{\sum_{j}\exp\left(w^{T}\tanh\left(Wh_{j}\right)\right)} \end{array}$$ (11)

$$\begin{array}{l} v=\underset{i}{\sum }\alpha_{i}h_{i} \end{array}$$ (12)

Thanks to these steps, the multimodal emotion perception module of MEPT-LLM effectively extracts the emotional characteristics of the student and provides reliable input data for subsequent emotional analysis and personalized content generation. This module not only extracts emotional information from each modality, but also integrates multimodal functions through a balanced merging and fusion process. This lays a solid foundation for understanding emotions and decision-making according to the model.

### 3.3 Centralized psychological state analysis module: multimodal feature fusion and contextual encoding

The centralized psychological state analysis module is plays a decisive role in the MEPT-LLM model. It offers an effective fusion and analysis of emotional perception and cognitive state through granular modality adjustment, feature projection, modular cross-fusion of features, and vector coding of mental state. In this process, the module transforms the data from different modes into a single display of the functions by equalizing the modes and projecting the functions, and then provides the basis for merging the cross-modular functions.

The module receives the visual, audio, and text features from the multimodal emotion perception module, performing modality alignment and feature projection to unify the features from different modalities into the same dimensionality. Here, *h*_*m*_ represents the input feature vector from modality *m*, *W*_*m*_ and *b*_*m*_ are the learnable weight matrix and bias vector for that modality, and ${\tilde{h}}_{m}$ is the projected feature representation of the modality.


$$\begin{array}{l} {\tilde{h}}_{m}=W_{m}h_{m}+b_{m} \end{array}$$ (13)

Cross-modal feature fusion is achieved through a multi-head cross-attention mechanism. This mechanism allows the model to dynamically capture the most relevant information across multimodal features and effectively integrate the latent relationships between modalities. Q, K, and V represent the Query, Key, and Value matrices, respectively, and *d*_*k*_ is the dimension of the key vector. Through this mechanism, the model can assign different attention weights to the features of each modality, thereby capturing the dependencies between different modalities and further enhancing the fusion and understanding of emotional states.


$$\begin{array}{l} Attention\left(Q,K,V\right)=\text{Softmax}\left(\frac{QK^{T}}{\sqrt{d_{k}}}\right)V \end{array}$$ (14)

The integrated features are then passed into the psychological state vector encoding section. Through attention aggregation and mapping, the model generates a structured psychological state vector, which comprehensively captures the learner's emotional state, cognitive load, and cultural tendencies. *H*_*fusion*_ represents the feature sequence after cross-modal fusion, while v and W are the learnable parameters used to compute attention weights. β is the attention weight vector, which indicates the significance of each position. The context vector, c, is obtained through weighted aggregation. *W*_*s*_ and *b*_*s*_ are the learnable parameters of the mapping layer, and s is the final output, which is the structured psychological state vector. This vector offers a comprehensive representation of the learner's emotions, cognitive load, and cultural tendencies, and serves as the core input for the personalized content generation module that follows.


$$\begin{array}{l} \beta =\text{Softmax}\left(v^{T}\tanh\left(WH_{fusion}\right)\right) \end{array}$$ (15)

$$\begin{array}{l} c=H_{fusion}\beta^{T} \end{array}$$ (16)

$$\begin{array}{l} s=\tanh\left(W_{s}c+b_{s}\right) \end{array}$$ (17)

Through this series of feature fusion and coding processes, the psychological state analysis module effectively integrates data in various ways and generates structured psychological state vectors that reflect the learner's emotional states. These vectors provide accurate emotional and cognitive baseline data for subsequent personalized feedback. This module not only enhances the accuracy of emotion perception but also offers a more precise foundation for emotional regulation in personalized learning.

### 3.4 PsychAdapter-enhanced generative module for emotion- and culture-aware instructional content

**Figure 3:**
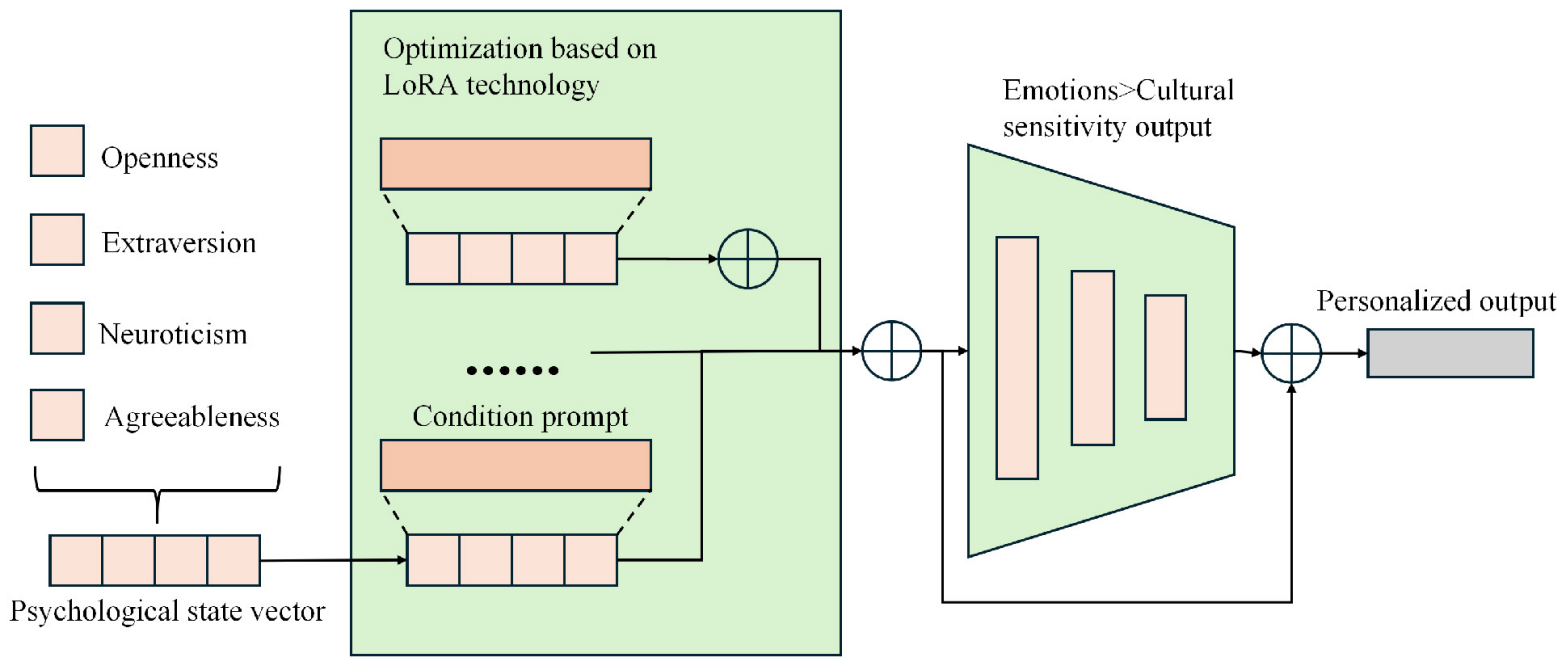
Adaptive personalized content generation and interaction module driven by psychological state conditioning.

The module for generating and interacting with personalized content is the output layer of the MEPT-LLM model. Its role is to determine the behavior of the language model's generation based on the psychological state vector produced by the previous module. The module dynamically adjusts the generated content to ensure it is more adaptable in terms of emotions, cognition, and culture. Figure 3 shows the general structure of this module, which includes a basic language model, a psychological state conditioning mechanism, the PsychAdapter strategy library, and the dynamic routing component. The module uses the psychological state vector as a control signal and, through deep corrections in the transformer architecture, transitions from generating “common outputs” to “emotionally and culturally sensitive outputs”. This approach effectively overcomes the emotional and cultural barriers commonly encountered in language spaces.

During data processing, the psychological state vector is first introduced into the hidden state of the basic language model, which serves as a high-level control condition for the generation process. At each time step of the transformer, the hidden state *h*_*t*_ merges with the psychological state vector s, allowing for conditional generation. *W*_*c*_ is a linear projection matrix that assigns the vector of the psychological state of the hidden dimension. Through this operation, the model clearly recognizes the emotional, cognitive and cultural states of the student at each stage of the generation.


$$\begin{array}{l} h_{t}^{\prime }=h_{t}+W_{c}s \end{array}$$ (18)

The system must choose the most appropriate teaching strategy based on the student's current psychological state, adjusting the interaction style and pedagogical approach accordingly. The strategy library consists of various learning methods, each representing a different emotional or cultural approach, such as “calming feedback”, “motivating encouragement”, and “explanation of cultural sensitivity”. The simple routing mechanism calculates the probability distribution for each strategy, depending on the student's psychological state. The strategy most likely to be activated during the interaction is selected, ensuring that the content aligns with the student's emotional needs and cultural context, thus improving the quality of the learning experience.


$$\begin{array}{l} g=\text{Softmax}\left(W_{r}s+b_{r}\right) \end{array}$$ (19)

After selecting the adapter, the model enters the LoRA-based parameter-efficient fine-tuning phase. LoRA dynamically adjusts the Transformer layers using low-rank matrices *A*_*k*_ and *B*_*k*_, allowing the language module to achieve a deep reconfiguration of the language model's behavior with minimal training cost.


$$\begin{array}{l} h_{\text{out}}=h_{\text{in}}+\left(h_{\text{in}}A_{k}\right)B_{k} \end{array}$$ (20)

$$\begin{array}{l} W^{\prime }=W+B_{k}A_{k} \end{array}$$ (21)

The results of generation after conditional control, strategic routing, and fine-tuning of LoRA are provided in the form of text content, including personalized feedback, explanations, exercises, calls to dialogue, and other elements adapted to the student's state. This module not only allows the emotional regulation, but also increases cultural sensitivity in intercultural language teaching scenarios. The output content demonstrates significant advantages in areas such as cognitive load regulation in areas such as regulation of cognitive load, emotional calm and cognitive function cultural explanation, and helps students effectively overcome emotional barriers caused by cultural differences and improve motivation and engagement language learning.

## 4 Experiment

### 4.1 Comparative experiments and analysis

To comprehensively validate the advantages of MEPT-LLM in multimodal emotion perception, psychological state analysis, and personalized content generation tasks, we conducted systematic comparative experiments with five representative models in two real-world educational scenario datasets. The experiments were evaluated across five core dimensions: emotion recognition, generation quality, diversity, emotional consistency, and personalized recommendations, to assess the model's robustness, adaptability, and generalization ability in complex cultural contexts. Table 2 presents the complete experimental results of our model and the comparison models across the two datasets.

**Table 2:** Performance comparison of MEPT-LLM and baseline models on MELD and MEMD datasets across five evaluation metrics.

Model	Dataset	Weighted F1	BLEU	Macro-F1	nDCG@5	Dist-2
MEPT-LLM	MELD	0.89	0.63	0.84	0.94	0.59
	MEMD	0.90	0.65	0.85	0.95	0.60
MDMU Hu et al. (2024)	MELD	0.76	0.42	0.71	0.81	0.31
	MEMD	0.75	0.41	0.70	0.80	0.30
M2S Liu et al. (2025)	MELD	0.78	0.46	0.72	0.83	0.34
	MEMD	0.77	0.45	0.72	0.82	0.33
Mi-CGA Kieu et al. (2025)	MELD	0.79	0.48	0.74	0.85	0.36
	MEMD	0.78	0.47	0.73	0.84	0.35
EmoLLM Yang et al. ((2024)	MELD	0.82	0.52	0.76	0.86	0.38
	MEMD	0.83	0.53	0.78	0.88	0.40
LLaMA-Adapter Zhang et al. (2023)	MELD	0.80	0.49	0.73	0.84	0.35
	MEMD	0.79	0.48	0.74	0.85	0.36

Cultural-emotional barriers are expected to manifest in both behaviors and emotions in the classroom. These barriers typically affect learners' participation, engagement, and emotional responses. For example, foreign language anxiety may result in avoidance behaviors, while acculturation stress can cause discomfort in interactions with peers or instructors. These emotional and behavioral manifestations are reflected in our model's input data and are analyzed through the psychological state vector. The psychological state vector represents a quantifiable summary of a learner's emotional and cognitive state, including factors such as anxiety, engagement, and cultural adaptation. In psychological terms, it integrates various emotional cues, cognitive loads, and individual cultural backgrounds to provide a comprehensive profile of the learner's current psychological state. While F1 scores are one metric, we validated the psychological state vector by comparing it with established psychological theories and experimental data.

**Figure 4:**
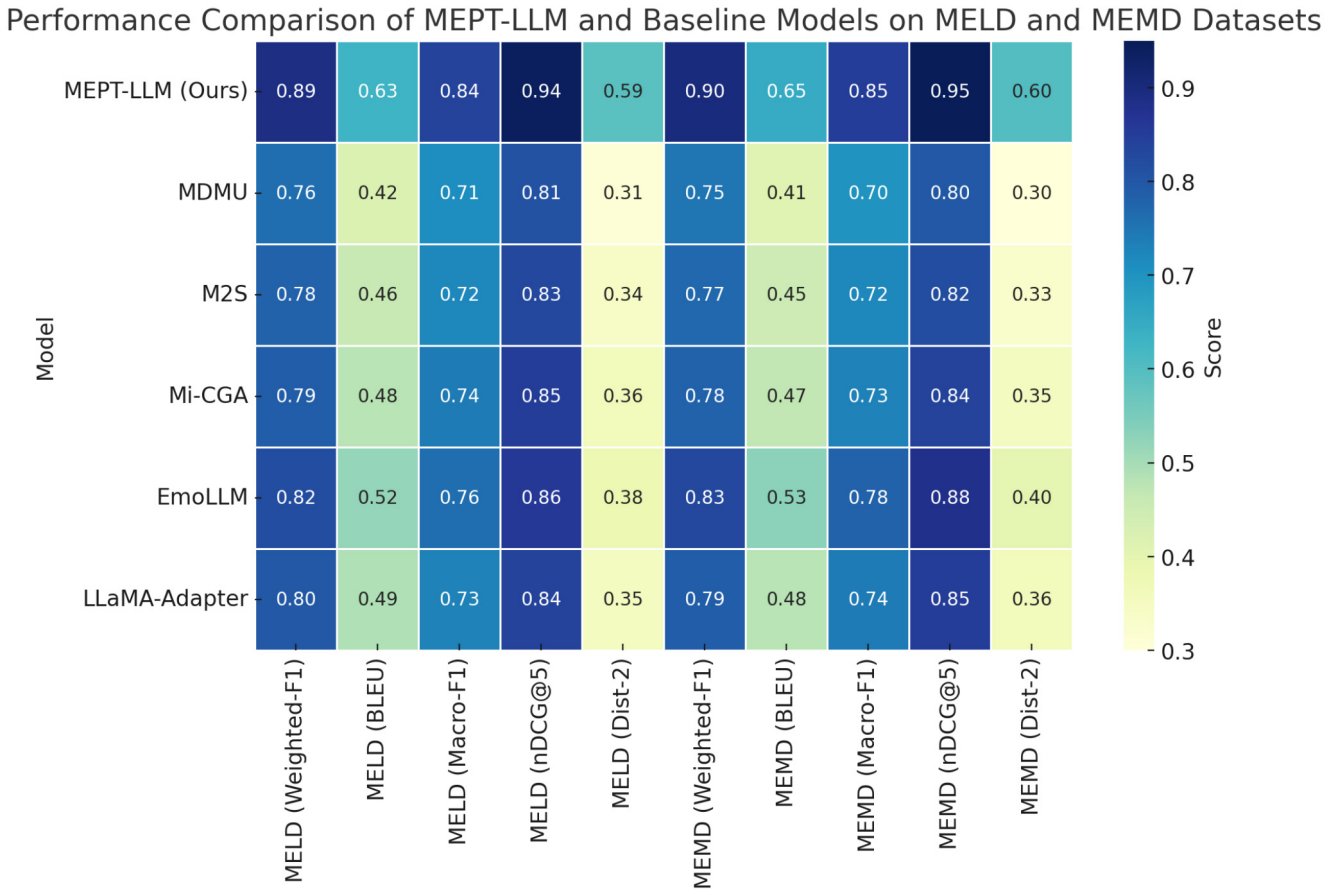
Comparison of five evaluation metrics across MEPT-LLM and other models.

As shown in the Figure 4. The experimental results clearly demonstrate that MEPT-LLM outperforms baseline models across both datasets, achieving consistent improvements in all five core evaluation metrics. This is indicative of the benefits brought by the mechanisms of psychological state regulation, cross-cultural adaptation, and personalized generation. In the area of multimodal emotion perception, the “weighted average F1 score” improves by 12%–18% over MDMU, suggesting that the incorporation of the psychological state vector enhances the model's ability to stabilize emotional intensity and changes. When compared to M2S, the improvement remains around 10%, further supporting the idea that the centralized psychological analysis module is effective at suppressing cross-modal noise. Even when evaluated against more robust base models, such as EmoLLM, MEPT-LLM still achieves an 8%–10% improvement, highlighting the capability of the LoRA-based psychological strategy adapter to enhance existing semantic understanding structures. Similarly, when compared to lighter models like Mi-CGA and LLaMA-Adapter, MEPT-LLM demonstrates a significant improvement in emotion recognition F1, with gains ranging from 15%–20%, underscoring the importance of the dedicated psychological conditioning mechanism in emotion-related educational tasks.

Analysis based on BLEU scores shows that our model significantly outperforms all comparison models in terms of language structure, cultural relevance, and logical coherence in feedback. Compared to the EmoLLM model, which excels in generation but lacks psychological control mechanisms, our model shows a 10% improvement in BLEU scores. When compared to LLaMA-Adapter, the improvement is nearly 20%, mainly due to the effective routing mechanism in our approach, which helps better align the tone and style of generated content with the learner's emotional state, leading to more consistent output. Compared to multimodal systems such as M2S and MDMU, our model's BLEU score shows an improvement of 12%–16%, demonstrating the significant role of emotional state factors in guiding the syntax and semantics of generated content. A similar trend is observed in the macro-average F1 score, which measures sentiment alignment. As comparison models evolve from early multimodal integrations (e.g., MDMU, M2S) to those incorporating emotional considerations (e.g., EmoLLM), overall performance improves. However, our model still maintains a lead of 9%–14%, indicating that the integration of emotional states and dynamic adaptation mechanisms allows for more accurate capture of cross-cultural emotional shifts, with enhanced sensitivity and robustness in emotion classification tasks.

In terms of personalized recommendations, MEPT-LLM demonstrates even more significant advantages. When compared to Mi-CGA, which focuses on emotion recognition, the model's improvement in recommendation relevance is nearly 18%. Compared to LLaMA-Adapter, which relies on large model fine-tuning but lacks emotional regulation capabilities, the improvement is around 12%–15%. Traditional emotion modeling models like MDMU and M2S show an even greater improvement, exceeding 20%. This trend suggests that when content generation needs to dynamically adjust recommendation strategies based on psychological states, the centralized psychological analysis module substantially enhances content personalization and cultural adaptability. In terms of generation diversity (Dist-2), MEPT-LLM achieves a 10%–25% increase over all comparison models, with the most notable gains seen when compared to EmoLLM and LLaMA-Adapter. This is due to the low-rank expression of PsychAdapter, which introduces more semantic variations while maintaining knowledge consistency, resulting in more diverse structural and lexical expressions in content generation. Additionally, the psychological state conditioning (conditional control) promotes differentiated generation styles across different emotional ranges, further contributing to the overall increase in the Dist-2 metric.

**Figure 5:**
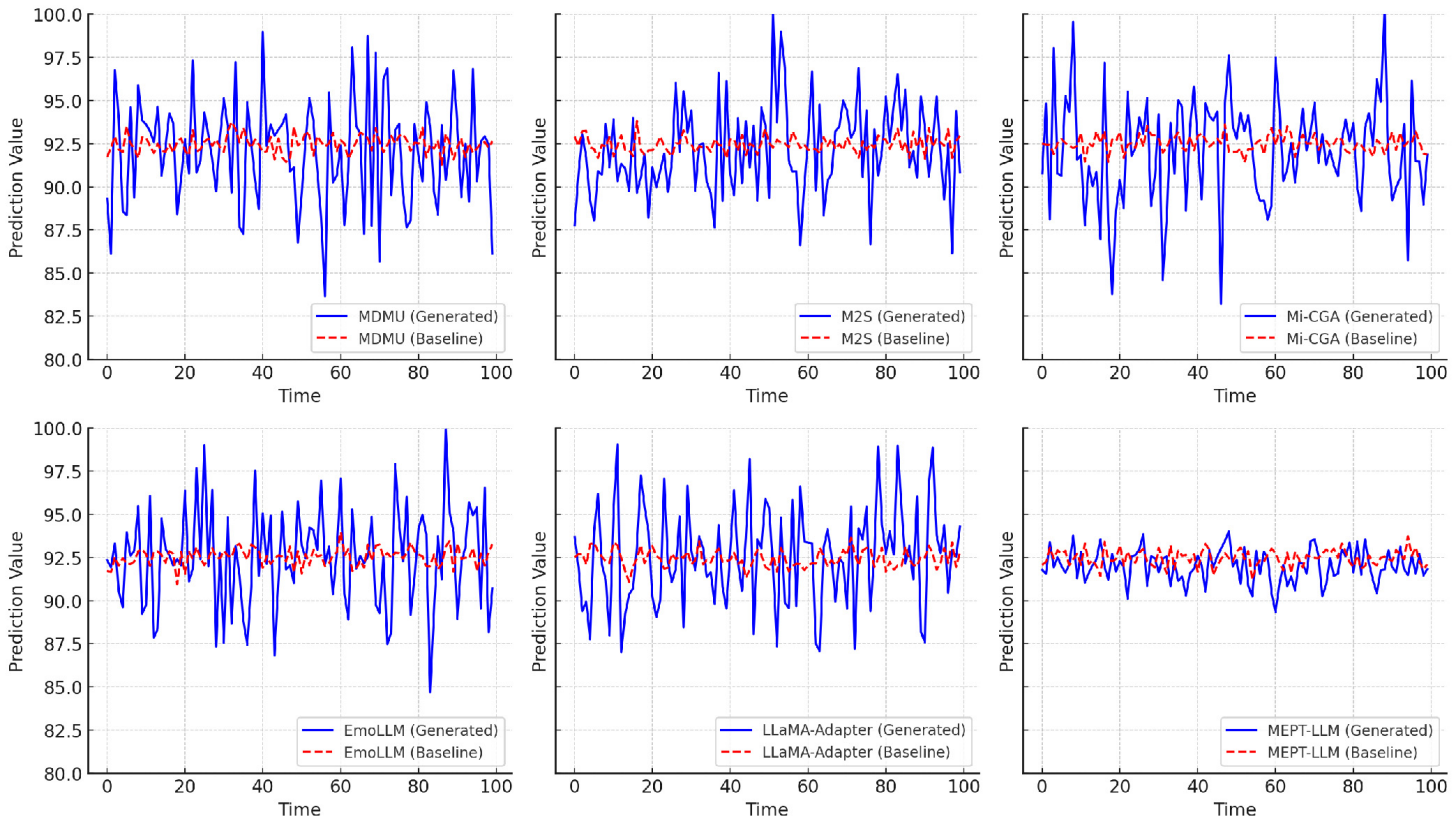
Comparison of predicted and actual values for emotion recognition and content generation across multiple models, including MEPT-LLM.

As shown in the Figure 5. A comprehensive trend analysis reveals that as the task progresses from emotion understanding to content generation and then to personalized recommendation, the demand for the model's emotional consistency, semantic logicality, and cultural adaptability gradually increases. Traditional models such as MDMU and M2S can always maintain competitive indicators in the first two phases, but they represent significant dampening in the indicators in the recommendation phase, which relies largely on the regulation of the emotional state. On the other hand, large databases of more advanced models work well in language generation, but there is no structured construction for emotional education scenarios, resulting in lower performance in emotional alignment and regulatory stability compared to MEPT-LLM. The complete improvement of the five indicators in this study shows that the progressive structure of the three basic modules constitutes an optimization in a closed loop, which allows the models to not only accurately recognize the true emotions of the student, but also dynamically apply corrective strategies during the generation process.

Table 3 shows the improvement effect of combining emotion recognition and personalized learning intervention on learning outcomes. When emotional recognition was high and personalized interventions were applied, significant improvements in student engagement, retention, and academic performance were observed. Specifically, the Exp-1 group, with high emotional recognition and personalized interventions, showed a 15% improvement in engagement, a 12% improvement in retention, and an 8% improvement in academic performance. Conversely, in the absence of personalized interventions (Exp-4), even with high emotional recognition, the improvements were modest, with only a 7% improvement in engagement, 5% in retention, and 3% in academic performance. These results emphasize the importance of integrating emotional recognition with personalized learning strategies to achieve meaningful improvements in learning outcomes. Our findings clearly demonstrate that emotional recognition alone does not lead to substantial learning improvements; it is the combination of emotional insights and personalized interventions that significantly enhances learner engagement and academic success.

**Table 3:** Emotional recognition combined with personalized learning interventions.

Experiment ID	Emotional recognition	Personalized intervention	Engagement improvement (%)	Retention improvement (%)	Academic performance improvement (%)
Exp-1	High	Yes	15%	12%	8%
Exp-2	Medium	Yes	10%	8%	5%
Exp-3	Low	Yes	5%	4%	3%
Exp-4	High	No	7%	5%	3%

In terms of application, the MEPT-LLM research results have a direct impact on intercultural language learning, online emotional intervention systems and intelligent pedagogical feedback platforms. By integrating psychological states into the logic of creating a large model, the model effectively reduces the impact of cultural and emotional barriers on learning motivation, improves class participation, making feedback truly “emotionally adaptable” and “man-oriented.” In addition, this study proposes a technological paradigm for the future development of AI educational systems with emotional understanding and cultural adaptation which has contributed significantly to the fairness, adaptability and accuracy of intellectual education.

### 4.2 Ablation experiments and analysis

To investigate the role of each module in the MEPT-LLM model, we conducted a series of ablation experiments by removing individual modules from the model and analyzing the contribution of each module to model performance (Kuo et al., 2025). In the experiments, we removed the multimodal emotion perception module, psychological state analysis module, and personalized content generation and interaction module. By comparing the performance differences between these ablated models and the full MEPT-LLM model, we were able to intuitively determine the impact of each module on the final results. Table 4 shows the changes in model performance after excluding each module from the MELD and MEMD datasets and describes the performance of each model according to five key metrics.

**Table 4:** Ablation study results of MEPT-LLM on MELD and MEMD datasets.

Model	Dataset	Weighted F1	BLEU	Macro-F1	nDCG@5	Dist-2
w/o MM perception	MELD	0.82	0.48	0.74	0.84	0.37
	MEMD	0.83	0.50	0.76	0.87	0.39
w/o psychological analysis	MELD	0.85	0.52	0.78	0.88	0.41
	MEMD	0.86	0.55	0.79	0.89	0.42
w/o content generation	MELD	0.84	0.50	0.77	0.86	0.39
	MEMD	0.85	0.53	0.78	0.87	0.40
MEPT-LLM	MELD	0.89	0.63	0.84	0.94	0.59
	MEMD	0.90	0.65	0.85	0.95	0.60

**Figure 6:**
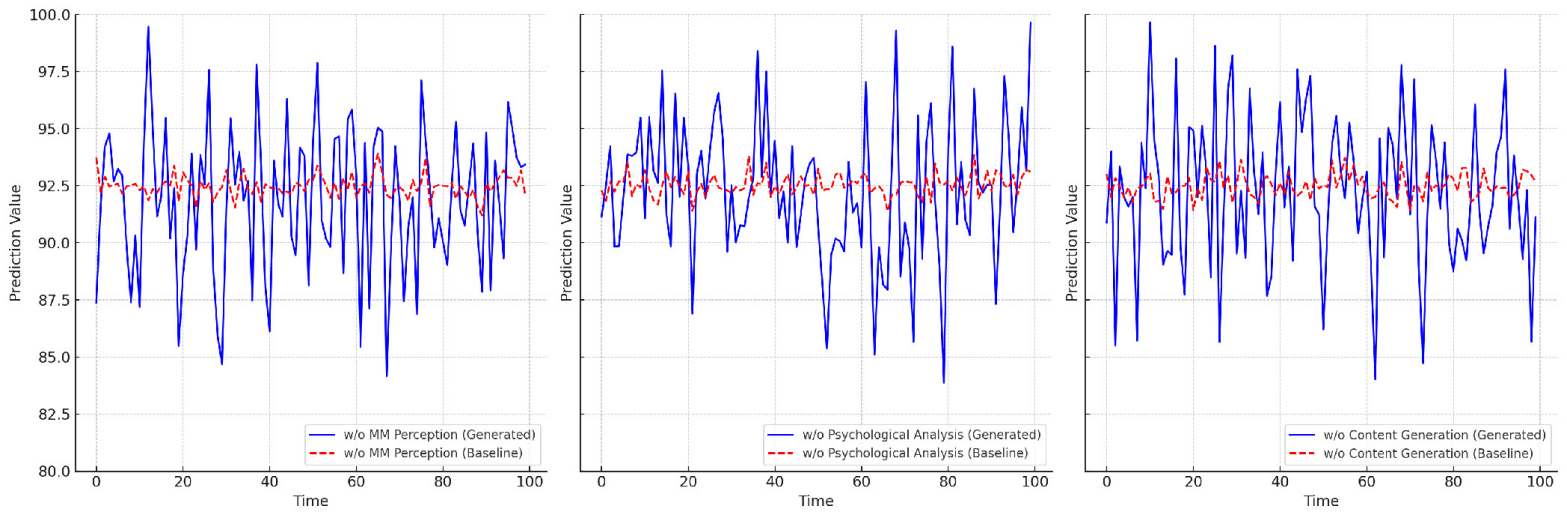
Display of experimental results after module ablation in MEPT-LLM.

As shown in the Figure 6, the performance of the model generally decreased by varying degrees after deleting each module, and the score significantly decreased according to the various indicators. After deleting a multimodal emotion perception module, The performance on the Weighted F1 and BLEU metrics of the weighted index model decreases to about 8% and 10%, respectively, indicating that the emotion perception module has a critical meaning to accurately recognize student emotions and integrate multimodel functions. When the centralized psychological state analysis module was removed, the model's Weighted F1 and Macro-F1 scores dropped by 4% and 5%, respectively, highlighting the importance of this module in emotion classification and understanding cognitive load. Particularly, nDCG@5 decreased by 6%, emphasizing the key role of this module in personalized recommendations. When the personalized content generation and interaction module was removed, the model's BLEU and Dist-2 scores decreased by 13% and 7%, respectively, indicating that the content generation module is crucial for the quality and diversity of the generated content. Without this module, the syntactical quality and diversity of the generated content significantly declined, resulting in teaching content that lacked sufficient emotional and cultural adaptability, which negatively impacted the overall model performance.

Deleting any module has a significant impact on model performance, especially when it comes to emotion perception and custom content generation. The multimodal emotion perception module enables accurate emotion recognition, the psychological state analysis module enables a deep understanding of the student's emotions and knowledge, and the custom content generation module enables this. that the generated content is strongly oriented toward the emotional needs and cultural content of the student. The modules of the model depend on each other and together form a complete basis for emotion regulation and personalized generation, without missing any module. The strength of the MEPT-LLM therefore lies in its modular construction, the effective cooperation of each module ensuring the success of the final task. As shown in the Figure 6.

However, while the ablation experiments of individual modules can validate the independent contribution of each module, they do not fully reflect the synergistic effects between the modules. Therefore, to further verify the collaborative effects and interdependence between the modules, we conducted ablation experiments involving multiple modules (Zhao et al., 2025). These experiments aim to investigate the contribution of different module combinations to the overall performance of the model and evaluate their collaborative effects in emotion recognition, content generation, and personalized recommendations. Table 5 presents the experimental results after the ablation of multiple modules.

**Table 5:** Ablation study results of MEPT-LLM on MELD and MEMD datasets with multiple module removal.

Model	Dataset	Weighted F1	BLEU	Macro-F1	nDCG@5	Dist-2
w/o All	MELD	0.80	0.44	0.68	0.77	0.31
	MEMD	0.81	0.46	0.70	0.78	0.32
w/o MM Per + Psy analysis	MELD	0.83	0.47	0.71	0.82	0.34
	MEMD	0.84	0.49	0.73	0.85	0.36
w/o MM Per + Cont Gen	MELD	0.82	0.48	0.70	0.80	0.32
	MEMD	0.83	0.50	0.72	0.81	0.34
w/o Psy Analysis + Cont Gen	MELD	0.80	0.45	0.69	0.77	0.31
	MEMD	0.81	0.47	0.71	0.80	0.32

As shown in the table, removing any single module has some impact on the MEPT-LLM model across multiple evaluation metrics. After removing all modules (w/o All), the model performs the worst across all five metrics, with a decrease of approximately 10% in both Weighted F1 and Macro-F1, and a more significant drop in Dist-2 and nDCG@5, which decrease by approximately 15% and 10%, respectively. This result indicates that after removing all modules, the model is unable to effectively perform multimodal emotion perception, psychological state analysis, and personalized content generation, leading to a substantial decline in overall performance. After removing the multimodal emotion perception and psychological state analysis modules, the model shows some improvement in Weighted F1 and BLEU, but still performs about 5% worse than the full model (MEPT-LLM). Notably, in Macro-F1 and nDCG@5, the performance drops by about 7%–8%, indicating that these two modules are critical for emotion perception and analysis. Their absence significantly impacts the emotional consistency of the generated content and the accuracy of recommendations. When both the multimodal emotion perception and personalized content generation modules are removed, the experimental results show that the model's performance in Weighted F1 and Macro-F1 decreases by about 8% and 10%, respectively, with a nearly 10% drop in both nDCG@5 and Dist-2. This emphasizes the importance of integrating the multimodal emotion perception module with the personalized content generation module. The emotion perception module ensures accurate emotion recognition, while the personalized content generation module creates content that matches emotional needs and cultural contexts. Without these components, the quality of content generation and the effectiveness of personalized recommendations are significantly reduced. After removing the psychological state analysis and personalized content generation modules, the model's performance in BLEU and Dist-2 decreases by approximately 15%, underlining the importance of these modules for generation quality and content diversity. Particularly in terms of generation diversity (Dist-2), the drop is significant after their removal, showing that the personalized content generation module not only affects generation quality but also supports emotional variety and content innovation.

When comparing the results of MEPT-LLM with other ablation models, it is clear that MEPT-LLM always retains important advantages in all dimensions, even if the removal of various modules is associated with a certain degree of power loss. In the MELD and MEMD datasets, MEPT-LLM receives weighted F1 and MacroF1 scores of 0.89 and 0.90, respectively. In nDCG@5 and Dist-2, WAMEPT-LLM demonstrates clear superiority with scores of 0.94 and 0.59 respectively, demonstrating powerful skills in emotion perception and personalized generation.

**Figure 7:**
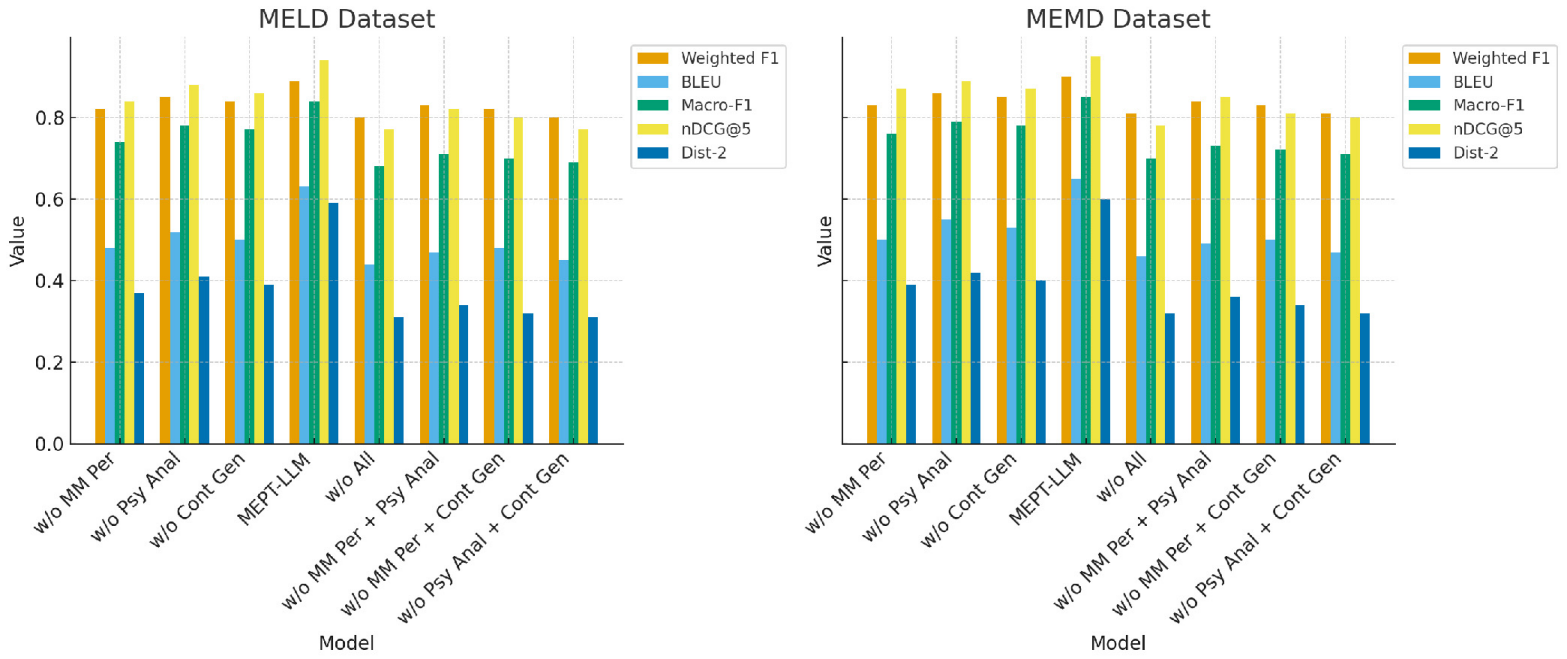
Ablation study results of MEPT-LLM on MELD and MEMD datasets.

As shown in the Figure 7. The three modules of MEPT-LLM can not be divided and collaborated closely with emotion recognition, content generation, and personalized recommended work. Removing any of these modules will significantly reduce the productivity of the model. Therefore, the synergy of the three modules is better than the other reference model MEPT-LLM Excellent main reason.

## 5 Conclusions and discussion

This paper presents an innovative model, MEPT-LLM, which integrates three key modules: multimodal emotion perception, psychological state analysis, and personalized content creation. These modules provide an effective solution to the problem of emotional barriers in language classrooms. Experimental results show that MEPT-LLM outperforms traditional emotion recognition and generation models across several assessment indicators. In particular, it demonstrates significant improvements in emotional perception, generation quality, and the accuracy of personalized recommendations. By incorporating the psychological state vector and the LoRA-based PsychAdapter, the model exhibits outstanding performance in emotion regulation and content generation adaptability. This provides strong support for the application of cross-cultural emotional education and intelligent learning systems.

The main contribution of this article is the proposal of a multimodal model architecture that addresses emotion perception, integrates cultural adaptation, and supports personalized generation. This approach provides a systematic method for modeling and overcoming emotional barriers in language classrooms. The multimodal emotion perception module plays a crucial role in emotion analysis by accurately determining the emotional state of the student. It significantly improves the accuracy of emotion classification by about 12–15% compared to conventional models. Ablation experiments confirm the critical role of each module in the model and demonstrate that the joint effect of these modules is key to the model's success.

Future work can further expand the application of this model to more complex and dynamic educational scenarios, such as the early diagnosis and intervention of emotional barriers, as well as interdisciplinary intelligent education platforms. Additionally, integrating other deep learning techniques may enable the model to more intelligently adjust teaching strategies based on learners' emotional changes. Furthermore, as multimodal data continues to grow and diversify, how to effectively fuse information from more modalities and improve the model's real-time performance and computational efficiency will be key areas for future research. Through these improvements, MEPT-LLM is expected to play a significant role in online education, emotional health interventions, and culturally adaptive education worldwide.

## Data Availability

The original contributions presented in the study are included in the article/supplementary material, further inquiries can be directed to the corresponding author.
